# A standardized postoperative bowel regimen protocol after spine surgery

**DOI:** 10.3389/fsurg.2023.1130223

**Published:** 2023-03-17

**Authors:** John K. Yue, Nishanth Krishnan, Albert S. Wang, Jason E. Chung, Leila L. Etemad, Geoffrey T. Manley, Phiroz E. Tarapore

**Affiliations:** ^1^ Department of Neurosurgery, University of California San Francisco, San Francisco, CA, United States; ^2^ Department of Neurosurgery, San Francisco Veterans Affairs Medical Center, San Francisco, CA, United States

**Keywords:** clinical protocol, cost effectiveness, gastrointestinal motility, ileus, postoperative care, quality of care, spine, standard of care

## Abstract

**Objectives:**

Spine surgery is associated with early impairment of gastrointestinal motility, with postoperative ileus rates of 5–12%. A standardized postoperative medication regimen aimed at early restoration of bowel function can reduce morbidity and cost, and its study should be prioritized.

**Methods:**

A standardized postoperative bowel medication protocol was implemented for all elective spine surgeries performed by a single neurosurgeon from March 1, 2022 to June 30, 2022 at a metropolitan Veterans Affairs medical center. Daily bowel function was tracked and medications were advanced using the protocol. Clinical, surgical, and length of stay data are reported.

**Results:**

Across 20 consecutive surgeries in 19 patients, mean age was 68.9 years [standard deviation (SD) = 10; range 40–84]. Seventy-four percent reported preoperative constipation. Surgeries consisted of 45% fusion and 55% decompression; lumbar retroperitoneal approaches constituted 30% (10% anterior, 20% lateral). Two patients were discharged in good condition prior to bowel movement after meeting institutional discharge criteria; the other 18 cases all had return of bowel function by postoperative day (POD) 3 (mean = 1.8-days, SD = 0.7). There were no inpatient or 30-day complications. Mean discharge occurred 3.3-days post-surgery (SD = 1.5; range 1–6; home 95%, skilled nursing facility 5%). Estimated cumulative cost of the bowel regimen was $17 on POD 3.

**Conclusions:**

Careful monitoring of return of bowel function after elective spine surgery is important for preventing ileus, reducing healthcare cost, and ensuring quality. Our standardized postoperative bowel regimen was associated with return of bowel function within 3 days and low costs. These findings can be utilized in quality-of-care pathways.

## Introduction

Spine deformity affects 30%–70% of the elderly population (1), and spine surgery for degenerative conditions has continued to rise over the past two decades (2–4). Optimizing outcomes and reducing complications in this growing population is paramount. Age-related factors, medical comorbidities, and perioperative opioid use can impact return of bowel function after spine surgery. Ileus, an extreme form of constipation associated with loss of bowel motility, usually manifests in 3–5 days after surgery (5–7), and constitutes a severe complication due to its morbidity, increased hospital length of stay (HLOS), and cost (8–10). Given rates of postoperative ileus at 5%–12% after spine surgery (11, 12), return of bowel function has become an important determinant of healthcare outcomes and cost.

The etiology of postoperative ileus is typically multifactorial. Risk factors include predisposing conditions (e.g., gastrointestinal disorders), perioperative fluid and electrolytes imbalance, stress and inflammation, and narcotic administration for pain control (13). In spine surgery, gastrointestinal morbidities are associated with fusion surgeries, longer intraoperative time, higher blood loss, and higher postoperative opioid doses (14). In addition, direct exposure and retraction of the abdominal viscera during anterior approaches to the thoracolumbar spine are thought to exacerbate postoperative bowel paresis and dysfunction (15). Ileus has been found to occur more frequently in patients undergoing ≥3-level posterior fusion (35% vs. 58%) (11). While not formally evaluated in spine surgery, the acute care impact of postoperative ileus has been extensively examined in abdominal surgery with significantly increased HLOS by 6 days (11.5 vs. 5.5 days) and per-patient costs by $9,000 ($18,877 vs. $9,460) (16).

The high cost of morbidity for postoperative ileus to both patients and healthcare institutions directly contrasts with the relatively low cost of prophylactic medications needed to improve gastrointestinal motility. While patients recovering from spine surgery often receive motility-promoting medications, standardized protocols for an optimized bowel regimen remain sparse. Recently, Enhanced Recovery After Surgery (ERAS) protocols have described general bowel regimens as part of integrated postoperative recovery (17, 18), however specific/stepwise management is rarely reported in detail. To date, only one report exists in the pediatric orthopedic literature regarding the implementation of a dedicated bowel regimen after spine surgery (19)^.^

Herein, we report findings from a single-institution study of elective spine surgery patients after implementation of a standardized bowel regimen protocol with specific details regarding tiered management. We discuss the nuances of careful monitoring for return of bowel function in tandem with our protocol in order to improve outcomes and cost.

## Materials and methods

### Rationale and patient selection

**Figure 1:**
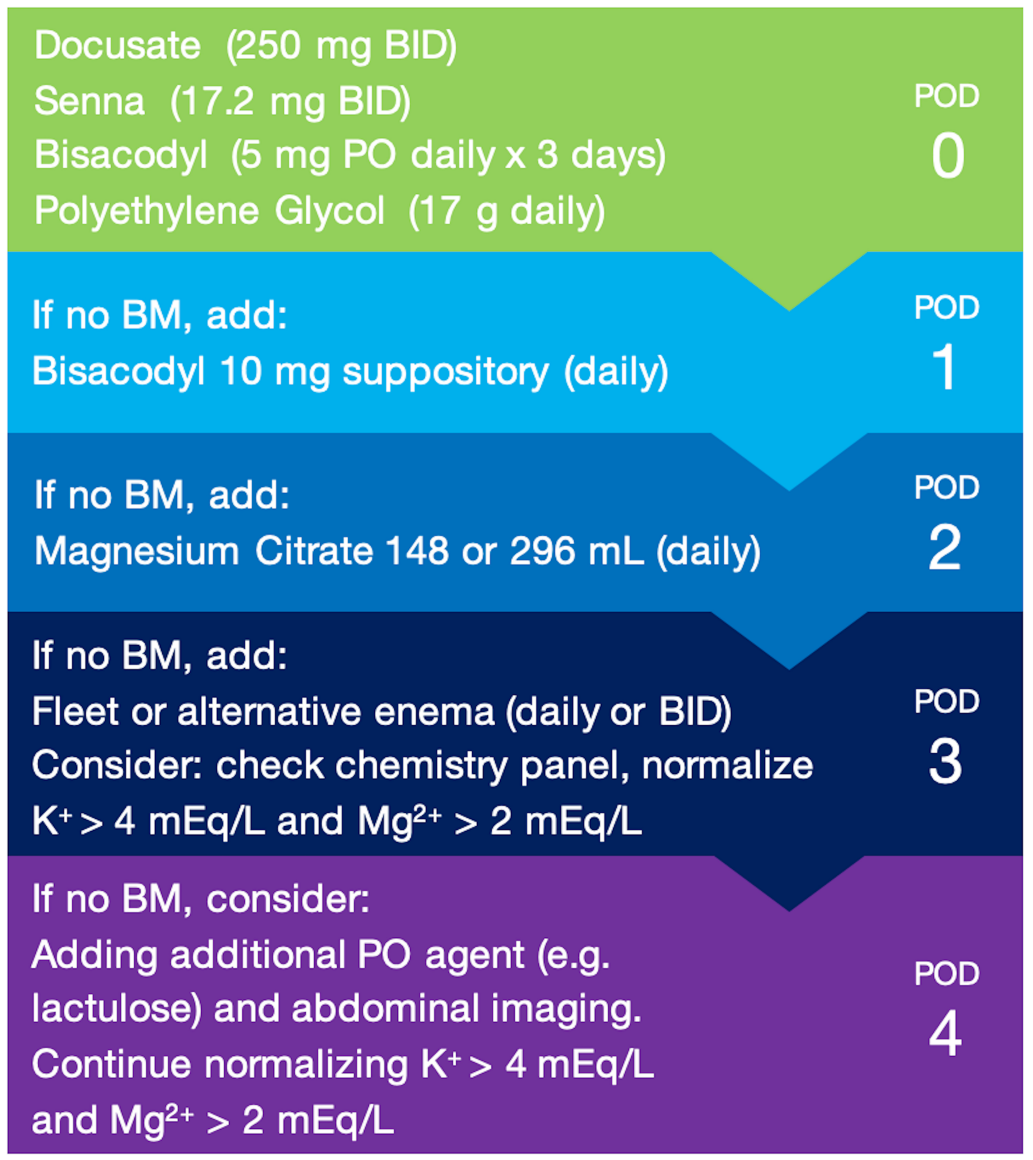
Standardized postoperative bowel regimen protocol. Standardized, tiered bowel regimen protocol implemented at our institution based on return of bowel function and postoperative day (POD). Additive medications beyond the POD 0 regimen should be withdrawn once the patient has return of bowel function. BID = twice per day; BM = bowel movement; PO = per oral (by mouth).

A standardized postoperative bowel medication protocol (Figure 1) was implemented as part of standard clinical care for all elective spine surgeries performed by a single neurosurgeon from March 1, 2022 to June 30, 2022 at a metropolitan Veterans Affairs medical center (San Francisco Veterans Affairs Medical Center (SFVAMC), California, United States (US)). The rationale for the implementation and close follow-up of return to bowel function at our institution originated from anecdotal observations of elective spine cases with persistent constipation and ileus. Further review revealed that practice variations existed in bowel medication administration and documentation of return of bowel function. As constipation and ileus have been reported after all types of spine surgery, the tiered bowel regimen protocol was implemented as part of standard care for our elective spine surgery patients. We did not restrict the current analysis by anatomic location (cervical/thoracic/lumbar), surgical approach (anterior/lateral/posterior), or technique (fusion/decompression). To focus on a more uniform cohort, patients who required emergent spine surgery and existing inpatients who developed an indication for spine surgery were not included in this analysis.

Our study was conducted in accordance with the Medical Association's Declaration of Helsinki, national and institutional guidelines. The study protocol was reviewed and approved by the University of California, San Francisco Committee on Human Research (UCSF CHR; study #22-37541), which is the SFVAMC Institutional Review Board of record. Our study consisted of retrospective medical record review without patient contact, and a waiver of informed consent was granted by the UCSF CHR.

### Standard multimodal postoperative care

All patients received standard of care multimodal pain management consisting of acetaminophen 1,000 mg every 6 h (Q6H), gabapentin 300 three times per day (TID), baclofen 5 mg twice per day (BID) as needed (PRN) for muscle spasms, oxycodone 2.5 mg Q4H PRN for moderate pain, oxycodone 5 mg Q4H PRN for severe pain, and hydromorphone 0.2 mg Q2H PRN for breakthrough pain. Patients sat up to chair for meals, underwent foley catheter removal on POD 1 morning, and underwent early mobility protocols including physical/occupational therapy starting POD 1. The exception was one patient who required a two-stage surgery on separate dates (stage 1: L4-S1 anterior interbody fusion; stage 2: L1-S1 posterior fusion with hardware revision 4 days later). He was mobilized in the interim and began physical/occupational therapy after completing his second surgery, in concordance with institutional guidelines. Standard dietary modifications at our institution include a clear liquid diet until bowel movement for anterior approach lumbar surgeries, and a soft diet until bowel movement for lateral approach lumbar surgeries, due to relatively increased risks of bowel paresis secondary to indirect manipulation from these retroperitoneal approaches. Once return of bowel function occurred, patients were advanced to a regular, heart-healthy diet.

### Standardized bowel regimen protocol

Daily bowel function was evaluated and bowel medications were advanced using the protocol as part of standard care. Starting POD 0, all patients received oral docusate 250 mg BID, oral senna 17.2 mg BID, and oral polyethylene glycol 17 g daily, and in addition, oral bisacodyl 5 mg daily for 3 days. On POD 1, bisacodyl suppository 10 mg was added to the regimen if bowel function had not returned. On POD 2, oral magnesium citrate 148 ml or 296 ml (per patient tolerance) was added to the regimen if bowel function had not returned. On POD 3, sodium phosphate (1 standard bottle of 118 ml contains 19 g monobasic sodium phosphate and 7 g dibasic sodium phosphate) or alternative enema daily or BID was added (per patient tolerance) if bowel function had not returned, a chemistry panel was sent, and electrolytes were repleted to normal values. If there was no return of bowel movement on POD 4, the existing protocol would be evaluated to consider an alternative oral agent (e.g., lactulose), in conjunction with a detailed clinical and abdominal exam, consideration of abdominal imaging, and appropriate specialist consultation for further evaluation (e.g., internal medicine, gastrointestinal, acute care surgery).

Our bowel regimen protocol was verified with institutional pharmacy guidelines to be within the daily safe maximum dosages for each medication. Once there was return of bowel function, the protocol reverted back to the POD 0 regimen of docusate/senna/polyethylene glycol, with oral bisacodyl if within 3 days post-surgery (per patient tolerance), and other additive medications were withdrawn.

### Pharmacology of bowel regimen medications

A feature of our protocol was the careful selection of pharmacological agents with distinct and complementary mechanisms of action. The medications utilized in the bowel regimen protocol fall into the categories of stool softeners (docusate), stimulant laxatives (senna, bisacodyl), and osmotic agents (polyethylene glycol, magnesium citrate, fleet enema). Docusate is an anionic surfactant that lowers the surface tension between the oil-water interface of stool, increasing water and lipid penetration of stool to facilitate excretion (20). Senna and bisacodyl act through direct contact with the submucosal and myenteric plexuses to increase intestinal motility and secretions, thereby increasing peristalsis (21). Polyethylene glycol, magnesium citrate, and sodium phosphate/fleet enema are osmotic preparations that draw water into the intestinal lumen to stimulate motility through increased volume (20).

### Statistical analysis

Detailed clinical, surgical, and postoperative data are reported, including American Society of Anesthesiologists Physical Status Classification (ASA), surgery indication/approach, postoperative care level, estimated intraoperative blood loss (EBL), complications, and discharge destination. As part of standard care, patients were queried on admission whether they had preoperative constipation, and daily postoperatively regarding whether they experienced discomfort attributable to their bowel regimen. Inpatient and 30-day complications were assessed by chart review. Day of return of bowel function and day of hospital discharge are reported.

As medication costs differ by institution, the estimated daily and cumulative costs of our bowel regimen protocol were calculated using two widely-utilized sources of medication pricing in the public domain. The Drugs.com database (https://www.drugs.com/price-guide/) is powered by several independent leading medical-information suppliers. GoodRx (https://www.goodrx.com) is a price comparison resource that aggregates prescription drug prices across 8–10 leading US pharmacies. Each medication was entered onto these two sources, and costs were calculated based on per-unit pricing; for GoodRx, costs were averaged across available leading pharmacies. We then calculated the average between Drugs.com and GoodRx to provide the final estimated price for each medication.

Descriptive statistics are reported as mean and standard deviation (SD) for continuous variables, and proportions for categorical variables. A limited exploratory analysis was conducted to identify factors that may be associated with time to return of bowel function. Age (divided into 10-year subgroups), history of constipation, ASA grade, surgical approach (cervical, posterior thoracolumbar, anterior or lateral thoracolumbar) (11, 15, 22), and EBL (0–299 ml vs. ≥300 ml; approximate volume of 1 unit of packed red blood cells) were considered, with days to first bowel movement as the dependent variable. Univariate linear regressions were performed, and variables statistically significant at *p* < 0.05 were entered onto a multivariable linear regression. Mean differences (*B*) and 95% confidence intervals (CI) are reported. Analyses were performed using Statistical Package for Social Sciences, version 29 (IBM Corporation, Armonk, NY, US).

## Results

### Baseline and surgical characteristics

Our study included 20 consecutive surgeries in 19 patients. One patient had a 2-stage surgery on different dates. Mean age was 68.9 years (SD 10; range 40–84), 94.7% were male, and 73.7% reported preoperative constipation. By ASA grade, 5.2% were ASA 1, 52.7% ASA 2, and 42.1% ASA 3. Detailed patient characteristics are presented in Table 1.

**Table 1:** Detailed characteristics of included patients.

Case	Age	Sex	Preoperative Constipation	ASA Grade	Pathology	Surgery	EBL	Admit Location	Surgical Drains	Complications	BM Day	Floor Day	Discharge Day	Discharge Destination
#1	78	M	No	2	Lumbar stenosis; DDD; neurogenic claudication	L3-5 posterior decompression	100	Ward	1	None	1	NA	4	Home
#2	56	M	No	2	Lumbar radiculopathy	L4 posterior decompression	200	Ward	1	None	1	NA	2	Home
#3	75	M	No	3	Cervical spondylotic myelopathy	C4-6 PSF with decompression	100	Ward	1	None	NA	NA	2	Home
#4	68	M	Yes	3	Lumbar stenosis; neurogenic claudication	L3-L5 posterior decompression	400	Ward	2	None	3	NA	6	Home
#5	71	M	Yes	3	Lumbar stenosis; DDD	L4-5 LLIF, PSF, with decompression	50	Ward	1	None	2	NA	3	Home
#6	63	M	Yes	2	Scoliosis; lumbar stenosis	L3-5 LLIF, PSF	100	Ward	0	None	1	NA	4	Home
#7	84	M	No	3	Thoracic disc herniation	T9-11 posterior decompression	50	Stepdown	1	None	1	NA	6	Skilled Nursing Facility
#8	78	M	Yes	2	Lumbar radiculopathy	L4-S1 posterior decompression	50	Ward	0	None	1	NA	3	Home
#9	62	M	Yes	2	Lumbar stenosis; DDD; neurogenic claudication	L4-S1 posterior decompression	50	Ward	1	None	2	NA	4	Home
#10	60	F	Yes	2	Lumbar stenosis; neurogenic claudication	L4-5 posterior decompression	30	Ward	1	None	2	NA	2	Home
#11	75	M	Yes	2	Cervical spondylotic myelopathy	C3-4 PSF with decompression	25	Stepdown	1	None	1	NA	2	Home
#12	40	M	No	1	Cervical spondylotic myelopathy	C4-6 ACDF	25	Ward	1	None	NA	NA	1	Home
#13	76	M	Yes	3	Scoliosis; lumbar stenosis; ASD, radiculopathy	L4-S1 ALIF	250	Stepdown	0	None	3	NA	NA	Underwent Stage 2 Surgery (Case #14)
#14	76	M	Yes	3	Scoliosis; lumbar stenosis; ASD, radiculopathy	L1-S1 revision PSF; L1-L2 LLIF	1000	Intensive Care Unit	2	None	3	2	6	Home
#15	68	M	Yes	3	Lumbar stenosis; neurogenic claudication	L4-5 posterior decompression	30	Ward	1	None	2	NA	3	Home
#16	78	M	Yes	2	Cervical spondylotic myelopathy	C4-6 laminoplasty	25	Ward	1	None	2	NA	4	Home
#17	74	M	Yes	3	Lumbar stenosis; DDD; neurogenic claudication	L3-4 posterior decompression	5	Ward	1	None	2	NA	3	Home
#18	67	M	Yes	2	Lumbar stenosis	L3-4 posterior decompression	100	Ward	1	None	2	NA	3	Home
#19	67	M	Yes	2	Lumbar stenosis, ASD	L3-4 LLIF	100	Ward	0	None	1	NA	2	Home
#20	70	M	Yes	2	Lumbar spondylolisthesis; radiculopathy	L5-S1 ALIF, PSF	100	Ward	0	None	2	NA	3	Home

Demographic, clinical, surgical, and length of stay characteristics. Days shown are postoperative days. Two patients (Case #3, #11) were discharged home in good condition prior to having a bowel movement. Case #13 and #14 were the same patient who underwent a 2-stage surgery 4 days apart during the same hospitalization. Spinal level is abbreviated (C, cervical; T, thoracic; L, lumbar). ACDF, anterior cervical discectomy and fusion; ASA, American Society of Anesthesiologists Physical Classification Score; ALIF, anterior lumbar interbody fusion; ASD, adjacent segment disease; BM, bowel movement; DDD, degenerative disc disease; EBL, estimated intraoperative blood loss; LLIF, lateral lumbar interbody fusion; NA, not applicable; PSF, posterior spinal fusion.

Surgeries consisted of 20% cervical (anterior fusion = 1, posterior fusion = 2, posterior decompression = 1), 5% thoracic (posterior decompression = 1), and 75% lumbar (anterior fusion = 1, combined anterior/posterior fusion = 1, lateral fusion = 1, combined lateral/posterior fusion = 3, posterior decompression = 9). Of the 6 lumbar fusion surgeries, 3 were one-level, 2 were two-level, and 1 was five-level. By EBL, 50% were 0–99 ml, 40% were 101–299 ml, and 10% were ≥300 ml. Ninety-five percent were admitted to floor and 5% to ICU.

### Postoperative course and return of bowel function

Eighteen patients had return of bowel function by POD 3 (mean 1.8 days, SD 0.7). Two patients were discharged in good condition after uncomplicated cervical fusion prior to bowel movement (Table 1; case #3, C4–6 posterior fusion and decompression; case #12, C4–6 anterior discectomy and fusion) as they reported no subjective complaints or gastrointestinal discomfort, were tolerating a regular diet with flatus, and met institutional discharge criteria on POD 2 and 1, respectively. Mean HLOS was 3.3 days post-surgery (SD 1.5, range 1–6). Overall, 94.7% (18 of 19 patients) were discharged to home, and 5.3% (1 of 19) to skilled nursing facility (SNF). There were no documented inpatient or 30-day complications, and no patient reported subjective discomfort due to their bowel regimen.

On exploratory analysis of the 18 patients with documented return of bowel function, history of constipation (No vs. Yes: mean 1.0 vs. 1.9 days; *p* = 0.040), ASA (Grade 2 vs. 3: 1.5 vs. 2.3 days; *p* < 0.001), and EBL (0–299 vs. ≥300 ml: 1.6 vs. 3.0 days; *p* = 0.008) were associated with time to return of bowel function on univariate analyses, while age and surgical approach were not (Supplementary Table S1). On multivariable analysis, history of constipation [*B*: + 0.8 days, 95% CI (0.1–1.4); *p* = 0.023] and ASA Grade 3 [*B: *+ 0.6 days (0.0–1.1); *p* = 0.049] remained statistically significant factors, while a nonsignificant statistical trend was observed for EBL ≥300 ml [*B: *+ 0.8 days (0.0–1.7); *p* = 0.057].

### Bowel regimen protocol cost analysis

**Figure 2:**
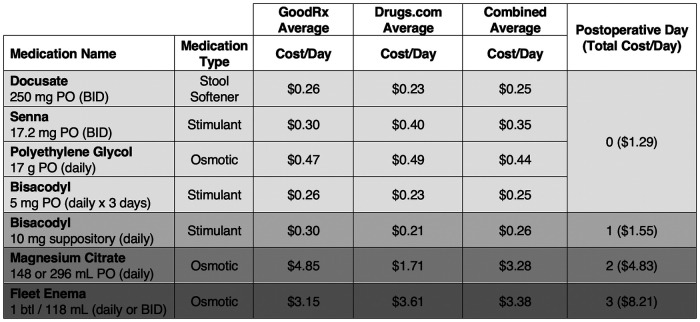
Estimated daily cost of postoperative bowel regimen protocol. Estimated costs of the standardized bowel regimen for each postoperative day were calculated using two widely utilized sources of medication pricing in the public domain: the Drugs.com database (https://www.drugs.com/price-guide/) and GoodRx (https://www.goodrx.com). $ = United States dollar; BID = twice per day; btl = bottle; PO = per oral (by mouth).

The estimated per-patient cost of the implemented bowel regimen was $1–2 on POD 0, $1–2 on POD 1, $5 on POD 2, and $9–13 on POD 3 (Figure 2). No patient required advancement of their bowel regimen protocol beyond POD 3, nor exceeded a total estimated bowel regimen medication cost of $35 (for discharge on POD 6).

## Discussion

Postoperative ileus is a common complication of spine surgery and carries significant morbidity. Patients who experience ileus require sustained medical management and monitoring, which can prolong hospitalization and increase healthcare costs. Ileus can compound deleterious effects, e.g., poor nutritional intake and increased risks of infection, which can be life-threatening and further impact recovery (23). Ensuring expeditious recovery of bowel function after spine surgery is therefore paramount to improving postoperative outcomes and reducing overall costs.

### Advantages of a standardized bowel regimen protocol

The bowel regimen described here promotes recovery of gastrointestinal motility through a tiered protocol utilizing additive, multimodal medications with distinct mechanisms of action. In our report, no patient experienced ileus despite the prevalence of risk factors such as older age, male gender, and anterior/lateral approaches (12, 22). Surgeries performed on patients in the cohort include a representative mixture of case types by level (cervical, thoracic, lumbar), approaches (anterior, posterior), and procedures (fusions, decompressions).

Features of this bowel regimen protocol ensure ease of implementation. All specified medications have a low side-effect profile and are easily administered orally, in pill or liquid form, or rectally with the assistance of care providers. These supportive features helped with obtaining a 100% regimen adherence rate for patients in the cohort. Moreover, these medications are available over the counter and are expected to be widely accessible in inpatient settings. The low technical barrier and high accessibility of the included medications support its implementation at other sites of care. Relative to the estimated per-patient cost of ileus, the total cost of bowel prophylaxis for all patients in our study was considerably less. A majority (63%) of patients were discharged on or prior to POD 3, which is the day the regimen reaches its maximum daily cost of $8.21. Indirect costs were likely minimized by the short average HLOS. Anecdotally speaking, if the ileus rate after spine surgery is estimated at 5% (1 in 20 cases) (11, 12), and the estimated average per-patient cost of our bowel regimen is $25 (for POD 3–4 discharge), the regimen's aggregate cost over 20 patients would be $500 to offset over $9,000 in cost per ileus (16).

A growing body of evidence is exploring the development and evaluation of standardized protocols to minimize postoperative gastrointestinal dysmotility. In 2022, Bansal et. al. described the application of ERAS protocols in spine surgery (24), which offers comprehensive guidance to prevent and reduce a range of potential postoperative complications. In our anecdotal experience, implementation of a bowel regimen requires considerable attention to detail. ERAS bowel regimens have limitations, as the pathway does not provide guidance on next steps if return of bowel function has not occurred after 48–72 h. Through meticulous tracking of our data after implementation, we found that patients tolerated the bowel regimen well without adverse events (e.g., loose bowel movements, abdominal discomfort). Our protocol also outlines that additive medications beyond docusate/senna/polyethylene glycol should be withdrawn once the patient has return of bowel function to reduce the risk of adverse effects and minimize unnecessary costs. With careful attention from providers and standardization of an optimized bowel regimen protocol, gastrointestinal complications can be increasingly prevented in order to improve HLOS, morbidity, cost, and patient satisfaction metrics.

While limited in scope and generalizability, our exploratory analysis identified preoperative constipation and higher ASA grade as multivariable factors associated with increased time to return of bowel function. Higher ASA grade is a known predictor of gastrointestinal dysfunction after spine surgery (14). History of preoperative constipation, however, is not routinely queried in spine surgery patients, nor part of preoperative documentation in most spine ERAS protocols (17, 18, 24). A 2015 study of 612 orthopedic surgery patients found that severe preoperative constipation (≥2 laxatives from different classes for ≥6 months) was an independent risk factor for postoperative ileus (25). While preliminary, our findings suggest that assessment of history of constipation has relevance in the early identification of patients at risk for postoperative gastrointestinal dysmotility.

### Limitations

Our study has several limitations. Our sample size was small and under the care of a single neurosurgeon in a well-resourced, tertiary referral hospital, which enabled close monitoring of every patient and access to all necessary medications. The demographic and comorbidity characteristics of our sample are representative of the unique patient population served by our metropolitan Veterans Affairs medical system, and may not be generalizable to care settings with less access to resources and greater demographic diversity. Observations from our study were drawn from an uncontrolled case series, which precludes definitive and generalizable conclusions. As stated previously, the multivariable regressions performed in our study were exploratory given our limited sample size, and require validation in larger cohorts. Patients undergoing emergency spine surgery and/or inpatients referred to neurosurgery non-electively were not included. Future studies that employ rigorous epidemiological study designs and ability to adjust for covariates and/or confounders would increase the power of our observations.

US medication pricing is complex and varies by care setting. Recent US and Canadian reports have shown increasing rates of prescription medication spending in both inpatient and outpatient settings (26–28). A US health insurance organization reported that for 10 specialty medications within the top 25 medications by 2019 Medicare Part B spending (9 intravenous immunotherapy agents, 1 intramuscular neuromuscular blocker), hospital and physician office charges were 108% and 22% higher, respectively, compared to specialty pharmacies (29). Our cost analysis was limited to publicly-available data, and likely reflects outpatient rather than true inpatient costs, which may be higher. However, the medications in our protocol are relatively inexpensive and readily available over the counter, and may not incur the same cost discrepancies as specialty medications.

## Conclusions

Careful monitoring of return of bowel function after elective spine surgery is important for preventing gastrointestinal symptoms and ileus, reducing healthcare cost, and ensuring quality of care. Our standardized postoperative bowel regimen protocol was associated with return of bowel function within 3 days and low costs. These findings can be utilized in quality of care and quality improvement pathways.

## Data Availability

The raw data supporting the conclusions of this article will be made available by the authors, without undue reservation.

## References

[1] Smith C Lamba N Ou Z Vo Q-A Araujo-Lama L Lim S The prevalence of complications associated with lumbar and thoracic spinal deformity surgery in the elderly population: a meta-analysis. J Spine Surg. (2019) 5:223–35. 10.21037/jss.2019.03.06 31380476 PMC6626743

[2] Grotle M Småstuen MC Fjeld O Grøvle L Helgeland J Storheim K Lumbar spine surgery across 15 years: trends, complications and reoperations in a longitudinal observational study from Norway. BMJ Open. (2019) 9:e028743. 10.1136/bmjopen-2018-028743 31375617 PMC6688683

[3] Rajaee SS Bae HW Kanim LEA Delamarter RB. Spinal fusion in the United States: analysis of trends from 1998 to 2008. Spine. (2012) 37:67–76. 10.1097/BRS.0b013e31820cccfb 21311399

[4] Sivasubramaniam V Patel HC Ozdemir BA Papadopoulos MC. Trends in hospital admissions and surgical procedures for degenerative lumbar spine disease in England: a 15-year time-series study. BMJ Open. (2015) 5:e009011. 10.1136/bmjopen-2015-009011 26671956 PMC4679892

[5] Venara A Neunlist M Slim K Barbieux J Colas PA Hamy A Postoperative ileus: pathophysiology, incidence, and prevention. J Visc Surg. (2016) 153:439–46. 10.1016/j.jviscsurg.2016.08.010 27666979

[6] Livingston EH Passaro EP Jr. Postoperative ileus. Dig Dis Sci. (1990) 35:121–32. 10.1007/BF01537233 2403907

[7] Holte K Kehlet H. Postoperative ileus: a preventable event. Br J Surg. (2000) 87:1480–93. 10.1046/j.1365-2168.2000.01595.x 11091234

[8] Khan M Joyce E Horn J Scoville JP Ravindra V Menacho ST. Postoperative bowel complications after non-shunt-related neurosurgical procedures: case series and review of the literature. Neurosurg Rev. (2022) 45:275–83. 10.1007/s10143-021-01609-y 34297261

[9] Jaber A Hemmer S Klotz R Ferbert T Hensel C Eisner C Bowel dysfunction after elective spinal surgery: etiology, diagnostics and management based on the medical literature and experience in a university hospital. Orthopade. (2021) 50:425–34. 10.1007/s00132-020-04034-z 33185695

[10] Oh CH Ji GY Yoon SH Hyun D Park H-C Kim YJ. Paralytic ileus and prophylactic gastrointestinal motility medication after spinal operation. Yonsei Med J. (2015) 56:1627–31. 10.3349/ymj.2015.56.6.1627 26446646 PMC4630052

[11] Yilmaz E Benca E Patel AP Hopkins S Blecher R Abdul-Jabbar A What are risk factors for an ileus after posterior spine surgery?-A case control study. Global Spine J. (2022) 12:1407–11. 10.1177/2192568220981971 33432832 PMC9393972

[12] Fineberg SJ Nandyala SV Kurd MF Marquez-Lara A Noureldin M Sankaranarayanan S Incidence and risk factors for postoperative ileus following anterior, posterior, and circumferential lumbar fusion. Spine J. (2014) 14:1680–5. 10.1016/j.spinee.2013.10.015 24184650

[13] Sindell S Causey MW Bradley T Poss M Moonka R Thirlby R. Expediting return of bowel function after colorectal surgery. Am J Surg. (2012) 203:644–8. 10.1016/j.amjsurg.2011.12.007 22459445

[14] Stienen MN Smoll NR Hildebrandt G Schaller K Tessitore E Gautschi OP. Constipation after thoraco-lumbar fusion surgery. Clin Neurol Neurosurg. (2014) 126:137–42. 10.1016/j.clineuro.2014.08.036 25255157

[15] Choy W Barrington N Garcia RM Kim RB Rodriguez H Lam S Risk factors for medical and surgical complications following single-level ALIF. Global Spine J. (2017) 7:141–7. 10.1177/2192568217694009 28507883 PMC5415155

[16] Goldstein JL Matuszewski KA Delaney CP Senagore A Chiao EF Shah M Inpatient economic burden of postoperative ileus associated with abdominal surgery in the United States. P T. (2007) 32:82–90. https://researchexperts.utmb.edu/en/publications/inpatient-economic-burden-of-postoperative-ileus-associated-with-.

[17] Porche K Samra R Melnick K Brennan M Vaziri S Seubert C Enhanced recovery after surgery (ERAS) for open transforaminal lumbar interbody fusion: a retrospective propensity-matched cohort study. Spine J. (2022) 22:399–410. 10.1016/j.spinee.2021.10.007 34687905 PMC9595392

[18] Debono B Corniola MV Pietton R Sabatier P Hamel O Tessitore E. Benefits of enhanced recovery after surgery for fusion in degenerative spine surgery: impact on outcome, length of stay, and patient satisfaction. Neurosurg Focus. (2019) 46:E6. 10.3171/2019.1.FOCUS18669 30933923

[19] Seilhamer C Di Lorenzo C Holstine J Samora JB. Creating a bowel management plan for pediatric orthopaedic spine surgery patients. Spine Deform. (2021) 9:365–71. 10.1007/s43390-020-00212-3 32978749

[20] Liu LWC. Chronic constipation: current treatment options. Can J Gastroenterol. (2011) 25(Suppl B):22B–8B. 10.1155/2011/360463 22114754 PMC3206558

[21] Tack J Müller-Lissner S. Treatment of chronic constipation: current pharmacologic approaches and future directions. Clin Gastroenterol Hepatol. (2009) 7:502–8. quiz 496. 10.1016/j.cgh.2008.12.006 19138759

[22] Park SC Chang SY Mok S Kim H Chang B-S Lee C-K. Risk factors for postoperative ileus after oblique lateral interbody fusion: a multivariate analysis. Spine J. (2021) 21:438–45. 10.1016/j.spinee.2020.10.002 33031922

[23] Weledji EP. Perspectives on paralytic ileus. Acute Med Surg. (2020) 7:e573. 10.1002/ams2.573 33024568 PMC7533151

[24] Bansal T Sharan AD Garg B. Enhanced recovery after surgery (ERAS) protocol in spine surgery. J Clin Orthop Trauma. (2022) 31:101944. 10.1016/j.jcot.2022.101944 35865326 PMC9293758

[25] Lee TH Lee JS Hong SJ Jang JY Jeon SR Byun DW Risk factors for postoperative ileus following orthopedic surgery: the role of chronic constipation. J Neurogastroenterol Motil. (2015) 21:121–5. 10.5056/jnm14077 25537675 PMC4288089

[26] Sherman M Curfman GD Parent J Wagner AK. Prescription medications account for one in four dollars spent by A commercial health plan. Health Affairs Forefront. (2018) 10.1377/forefront.20180821.820628

[27] Hofmeister M Sivakumar A Clement F Hayes KN Law M Guertin JR Trends in Canadian prescription drug purchasing: 2001-2020. J Pharm Policy Pract. (2022) 15:20. 10.1186/s40545-022-00420-4 35300714 PMC8928614

[28] Tichy EM Hoffman JM Suda KJ Rim MH Tadrous M Cuellar S National trends in prescription drug expenditures and projections for 2022. Am J Health Syst Pharm. (2022) 79:1158–72. 10.1093/ajhp/zxac102 35385103 PMC9383648

[29] New study: hospitals charge double for drugs—specialty pharmacies more affordable. AHIP. (2022). https://www.ahip.org/news/press-releases/new-study-hospitals-charge-double-for-drugs-specialty-pharmacies-more-affordable [Accessed February 16, 2023].

