# Supplementary material for: A standardized postoperative bowel regimen protocol after spine surgery
Source: Front Surg. 2023 Mar 17;10:1130223. doi: 10.3389/fsurg.2023.1130223 (PMC10063852; doi:10.3389/fsurg.2023.1130223)
Supplement: Supplementary file 1 [file Table1.pdf]

**Supplementary Table S1. Factors Associated with Time to Return of Bowel Function After Elective Spine Surgery**

| Variable | N | BM Day Mean (SD) | Univariate *B* [95% CI] | Multivariable *B* [95% CI] |
|---|---|---|---|---|
| ***Age*** | | | | |
| 56-64y | 4 | 1.5 (0.6) | --- | --- |
| 65-74y | 7 | 2.0 (0.6) | --- | --- |
| 75-84y | 7 | 1.7 (1.0) | --- | --- |
| ***PMH Constipation*** | | | | |
| No | 3 | 1.0 (0.0) | Reference | Reference |
| Yes | 15 | 1.9 (0.7) | 0.9 [0.1-1.8] | 0.8 [0.1-1.4] |
| ***ASA Grade*** | | | | |
| 2 | 11 | 1.5 (0.5) | Reference | Reference |
| 3 | 7 | 2.3 (0.8) | 0.8 [0.2-1.5] | 0.6 [0.0-1.1] |
| ***Surgery Type*** | | | | |
| Cervical, Posterior | 2 | 1.5 (0.7) | --- | --- |
| Thoracolumbar, Posterior | 10 | 1.7 (0.7) | --- | --- |
| Thoracolumbar, Anterior or Lateral | 6 | 2.0 (0.9) | --- | --- |
| ***Intraoperative EBL*** | | | | |
| 0-299 mL | 16 | 1.6 (0.6) | Reference | Reference |
| ≥300 mL | 2 | 3.0 (0.0) | 1.4 [0.4-2.3] | 0.8 [0.0-1.7] |

**Caption:** Factors associated with time to return of bowel function (BM Day) were evaluated in 18 cases with documented BM in-hospital. BM Day is defined as the first postoperative day of documented bowel movement, and means and standard deviations (SD) are shown for each variable. Univariate linear regressions were performed with BM Day as the dependent variable. Mean differences *(B)* and 95% confidence intervals (CI) are shown for variables with univariate comparisons statistically significant at $p<0.05$ [*B* is not shown for: Age (p=0.558); Surgery Type (p=0.649)]. The remaining variables were entered onto a multivariable linear regression. The multivariable p-values were: PMH Constipation (p=0.023), ASA Grade (p=0.049), Intraoperative EBL (p=0.057). ASA = American Society of Anesthesiologists Physical Status Classification; BM = bowel movement; EBL = estimated blood loss; PMH = prior medical history; y = years
